# Navigating the transition from early-stage investigator to mid-career: Challenges and opportunities for developing careers in diabetes translational research

**DOI:** 10.1017/cts.2026.10757

**Published:** 2026-06-04

**Authors:** Tainayah W. Thomas, Stephanie A. Hooker, Chibuzor Abasilim, Alyce S. Adams, Foster Osei Baah, Sarah S. Farabi, Julie Schmittdiel, Sarah A. Stotz, Luis A. Rodriguez

**Affiliations:** 1 Epidemiology and Population Health, Stanford University School of Medicine, Stanford, CA, USA; 2 Division of Research, Kaiser Permanente Northern California, Pleasanton, CA, USA; 3 Research and Evaluation Division, HealthPartners Institute, Minneapolis, MN, USA; 4 Division of Occupational and Environmental Health Sciences, School of Public Health, University of Illinois Chicago, Chicago, IL, USA; 5 Department of Epidemiology, University of Wisconsin–Milwaukee, Milwaukee, WI, USA; 6 Stanford Cancer Institute, Stanford University School of Medicine, Stanford, CA, USA; 7 Health Policy, Stanford University, School of Medicine, Stanford, CA, USA; 8 Emory University Nell Hodgson Woodruff School of Nursing, Atlanta, GA, USA; 9 Office of Nursing Research, Goldfarb School of Nursing at Barnes-Jewish College, St. Louis, MO, USA; 10 Food and Nutrition, Colorado State University, Fort Collins, CO, USA; 11 Kaiser Permanente Bernard J Tyson School of Medicine, Pasadena, CA, USA; 12 Department of Epidemiology & Biostatistics, University of California San Francisco, San Francisco, CA, USA

**Keywords:** Career development, early-stage investigators, translational research, enrichment programs, diabetes

## Abstract

Diabetes mellitus is a significant health burden, and recent reports indicate an increase in diabetes-related complications in the United States (U.S.). The mission of the National Institute of Diabetes and Digestive and Kidney Diseases Centers for Diabetes Translational Research (CDTR) is to improve the translation of research findings in diabetes prevention and treatment by supporting research across the translational science spectrum. To facilitate this mission, it is imperative to support multidisciplinary translational early-stage investigators (ESIs) to address the societal-, environmental-, and individual-level factors that impact diabetes outcomes. In this special communication article, we characterize the challenges, opportunities, and needs of ESIs transitioning to mid-careers whose goals are to develop a career in diabetes translational research. These challenges and opportunities were collected from 34 ESIs and 19 senior faculty members from 23 institutions across the U.S. who attended the inaugural CDTR National Enrichment Program Conference. Our findings aim to promote the engagement of ESIs in diabetes translational research and provide guidance in strengthening and sustaining their research careers.

## Introduction

Type 2 diabetes mellitus (T2 DM) is a significant health burden in the United States (U.S.), affecting over 38 million people and costing more than $327 billion annually [1, 2]. While the evidence base for the prevention and management of T2 DM is well-established, recent reports indicate an increase in diabetes complications in the U.S. [3]. Despite strong guidance for diabetes care, most patients do not achieve optimal treatment goals. For instance, the percentage of T2 DM patients meeting glycemic targets has remained around 50% or less over the past two decades [4]. Factors contributing to suboptimal outcomes in diabetes prevention and management include a complex interplay of individual, health system, societal, and policy-level influences, such as poverty, food insecurity, low educational attainment, limited health literacy, targeted marketing of unhealthy foods, and housing policies that restrict access to healthy food and safe physical activity environments [5, 6]. Limited investment and structural support for diabetes translational research have been repeatedly identified as barriers to achieving sustainable improvements in diabetes outcomes [7, 8].

Diabetes translational science identifies and addresses barriers to translating research into healthcare settings. Translational research seeks to improve healthcare by translating promising results from clinical studies into practice through action and change in real-world healthcare settings [9]. This includes health systems participatory research, dissemination and implementation research, pragmatic clinical trials, stakeholder-engaged research [10], and community-engaged participatory research. These stakeholder-engaged and participatory approaches increase the likelihood that research questions will be relevant, leading to scalable and sustainable diabetes treatment and prevention interventions while incorporating stakeholder needs and preferences [9]. Given these methods, diabetes translational researchers are well-positioned to evaluate and address unmet needs that could help alleviate suboptimal diabetes outcomes.

However, the pathway to support early-stage investigators (ESIs) in diabetes translational research remains unclear, with multiple factors influencing their career trajectories and success. Documented challenges for ESIs include lack of mentorship, insufficient institutional support, uncertainty in career pathways, limited NIH funding, inadequate support from public health and policy leaders, and social and environmental barriers [11]. These challenges may be particularly acute for ESIs in diabetes translational research, as traditional academic structures often do not incentivize health system and participatory research aimed at translating findings into action [12, 13].

The purpose of this Special Communication is to share insights from the inaugural Centers for Diabetes Translation Research National Enrichment Program that characterize the challenges, opportunities, and needs of ESIs transitioning to mid-careers in diabetes translational research from the perspective of ESI diabetes translational researchers. Our goal was to gather and highlight practice-informed insights from early-stage investigators and senior mentors to enhance academic environments and enable career success in diabetes translational research.

## Centers for diabetes translation research

The National Institute of Diabetes and Digestive and Kidney Diseases (NIDDK)-funded Centers for Diabetes Translation Research (CDTRs) program aims to improve translation of diabetes prevention and treatment research findings by supporting research across the translational research spectrum [14]. In 2023, NIDDK released a call for proposals for a program supplement to develop the National Enrichment Program. The Diabetes Research and Engagement through Advanced Multilevel Science Center for Diabetes Translational Research (DREAMS-CDTR, Grant#: P30DK092924), led by Stanford University, the University of California San Francisco, and Kaiser Permanente Division of Research, was awarded this supplement and hosted the inaugural National Enrichment Program (NEP).

### National enrichment program

The aims of the NEP were to: (1) create a “community of practice” of ESIs pursuing careers in diabetes translational research; (2) organize and conduct a national meeting of ESIs focused on a cutting-edge theme in diabetes translational research; and (3) disseminate summary findings from the national meeting, which was held in Redwood City, CA from June 5^th^–7^th^, 2024. This national meeting brought together 34 ESIs and 19 senior faculty members from across the United States representing 23 academic and research institutions. Three NIDDK program officials also attended the meeting.

## Methods

This report summarizes insights and recommendations generated during a structured conference session and is not designed or intended as a qualitative research study. To gather insights from ESIs, we conducted a conference session titled “What do your institutions, mentors, NIH, and public health and policy leaders need to do to help you succeed in diabetes translational research?” The perspectives, experiences, and knowledge shared during this session reflect insights from over 50 diabetes translational researchers on what ESIs need from five areas of support: mentors, the NIH, institutions, public health leaders, and policy leaders. The ninety-minute session was moderated by the lead and senior authors (Thomas and Rodriguez). The 54 attendees were divided into 10 tables for structured discussions, each focusing on one area of the question: “What do your <insert area> need to do to help you succeed and promote diabetes translational research?” Two tables were assigned to each area: institutions, NIH, mentors, public health leaders, and policy leaders. Each table included a mix of ESIs and senior mentors, with 20 minutes for discussion followed by a 5-minute report-back period for each table. After reporting back, there was a 15-minute open discussion. Two note-takers recorded detailed notes on the discussions. All participants consented to share their perspectives in this publication. Demographic information on attendees was not collected. Attendees included NIH defined ESIs – primarily post doctoral fellows and Assistant Professors – along with senior faculty mentors who contributed to the structured discussions.

## Development of findings

All conference attendees were invited to participate in manuscript development, and those who expressed interest were included as co-authors. The lead and senior authors held a kickoff meeting, sharing detailed discussion notes and a summary outline. Co-authors reviewed these materials for accuracy and collaboratively developed a writing plan. Each co-author drafted findings based on the topic corresponding to their structured conference session (i.e., institutions, NIH, mentors, public health leaders, and policy leaders). Drafts were informed by session notes, co-authors’ reflections, and their expertise as ESIs in diabetes translational research. To build consensus, all drafts were compiled into a single document for iterative review and editing. A second co-author meeting was then held to discuss, refine, and finalize the recommendations presented in the Discussion and Table 1.

**Table 1.** Challenges and opportunities for how institutions, mentors, the NIH, public health, and policy leaders can support early-stage investigators succeed in diabetes translational research Table 1 long description.

Area of support	Key challenges and opportunities	Existing opportunities or recommendations for needed programs informed by ESI discussions
**Institutions**	Commitment to providing comprehensive support for ESIs	Increased funding and livable stipends; expanding funding and support, especially for projects with short timelines Building bridges with IRB and research offices for ESI projects
	Encourage independent ideas and interests	Promote fresh perspectives using institutional communication platforms Spaces to explore ideas and develop skills
	Provide support during critical life events	Implement family-friendly initiatives and practices such as tenure clock extensions
	Facilitate ESI networking and cross-campus collaboration	Formal mentorship programs within and across organization ESI networks for diabetes translational research Assistance navigating research and organizational structures
	Transparency in processes to support fairness at all levels	Adopt best practices for fair and transparent recruitment, retention, and promotion of ESIs
	Advance community-engaged research and collaborations	Facilitate and streamline ESI collaboration and community-engaged research
**Mentors**	Help expand the network and grow career opportunities	Introduce mentees to senior diabetes translational researchers Recommend ESI for speaking engagements
	Provide emotional support	Normalize rejection and life events (e.g., starting a family)
	Provide role modeling	Have an open-door policy (as possible) on meetings with individuals like NIH program officers
	Support developing a mentorship team to meet multifaceted needs	Encourage ESI to find individuals who can help offer scientific advice and another who can provide emotional support
**NIH**	Increase pilot funding for ESI research	Increase funding to CDTRs for P&F award mechanism
	Support ESI growth and training	Support training workshops and opportunities focused on research skill development and diabetes translational research
	Increase visibility and knowledge about applying for NIH grants and options for leave	Hold public webinars about new funding opportunities and strategies for writing successful grants
	Support community-engaged research, recognizing the time and commitment of both researchers and community partners	Create funding opportunities to support engagement in research, reducing barriers for community partners
	Support multidisciplinary and cross-institutional partnerships, acknowledging the increased costs associated with quality team science	Offer funding opportunities for team science that encourage multidisciplinary collaboration and ESI leadership and have larger budgets to support cross-institutional partnerships
**Public health and policy leaders**	Improve access to public health datasets	Waive costs of fee-based datasets for ESIs
	Support ESIs’ work-life balance	Advocate for support for ESIs to have access to child and elder care
	Center community engagement	Provide networking opportunities with community-based organizations and hold community listening sessions for ESIs to recognize the importance of new ideas and perspectives
	Mentorship	Provide public health leadership with salary support to mentor ESIs
	Research environment	Create research environments for ESIs to succeed and thrive
	Career transition programs	Lobby for targeted funding mechanisms coupled with free and easy research data access to kick-start ESI-specific diabetes translational research
	Support legislation to enhance ESI research networks	Support partnerships and networks between ESIs across research institutions Establish institution-community partnerships that link ESIs to community leaders/champions

CDTR = centers for diabetes translational research; ESI = early-stage investigator; NIH = National Institutes of Health; P&F = pilot and feasibilty.

## Findings

Through structured discussions among ESIs and senior mentors, several key themes emerged regarding the support needed from institutions, mentors, the NIH, public health leaders, and policy leaders (Table 1).

### Research institutions

Institutions have significant influence and resources and are uniquely positioned to facilitate relationships between ESIs, mentors, funders, and community organizations involved in diabetes translational research. They can enhance ESI scientific and career advancement through comprehensive support, promoting independent ideas, offering resources during critical life events, facilitating networking and collaboration, ensuring transparency in hiring and promotion processes, and advancing community-engaged research.

#### Commitment to comprehensive support

Institutions should prioritize engagement with issues that directly impact ESI training, career development activities, and research. This should intersect with their advocacy efforts targeted at policymakers and funders. Support can include increased funding for graduate students and postdoctoral researchers focused on diabetes translational research, providing livable stipends to minimize financial strain. Expanded funding, including travel awards, seed funds for community engagement, and grants for short-term projects, is essential. ESIs suggest institutions help bridge gaps with institutional review boards and research offices to streamline translational and community-engagement research processes [15]. Institutions can also provide start-up packages for new ESI faculty, including salary support and research funds for preliminary data collection for career development (e.g., NIH K series) and R01 (or equivalent) applications. Ensuring transparent and fair recruitment, retention, promotion, and funding practices is also crucial.

#### Encouraging independent ideas

Science thrives on creative thinking and novel investigations. ESIs often have fully formed ideas that differ from existing institutional research or their mentors’ funded projects. Institutions should leverage communication platforms to promote fresh perspectives from ESIs. Institutional buy-in through scientific discovery and innovation funds can advance novel research ideas. For instance, ESIs suggest creating spaces for exploring diabetes translational research ideas through social events, journal clubs, and work-in-progress sessions, helping develop essential leadership, problem-solving, and communication skills.

#### Support during critical life events

Navigating personal responsibilities while pursuing research can be challenging. ESIs suggest institutions normalize the occurrence of personal events and hardships, ensuring they do not hinder career advancement. Institutions should promote family-friendly initiatives, such as the University of California’s Pay for Family Care and Bonding and Faculty Family Friendly Edge projects [16, 17]. Supporting practices like tenure clock extensions is particularly relevant, as translational research may require longer timelines than traditional biomedical or epidemiologic research, a challenge noted in national strategic reports on diabetes research and care [7].

#### Facilitating networking and collaboration

ESIs may experience isolation, hindering career advancement and research productivity, particularly in diabetes translational research where ESIs could be across multiple university departments (e.g., sociology, psychology) and schools (e.g., medicine, public health, social work). Institutions can foster formal networks for mentorship, connect ESIs within and across organizations, and advocate for communities that support ESI research and career development. Institutional and regional diabetes research centers may facilitate mentorship programs, collaborations, and multidisciplinary partnerships, enhancing understanding of institutional culture and supporting research dissemination.

#### Advancing community-engaged research

Community-engaged research requires collaboration with community partners. Institutions can develop infrastructure to support partnership development. To enhance ESI research impact, institutions should facilitate collaboration with community organizations. ESIs propose prioritizing community engagement through sound stewardship and providing resources grounded in community experiences. Institutional Clinical and Translational Science Awards (CTSAs) can support community-engaged research, though their reach may be limited to academic medical centers [18].

### Mentors

Mentors can expand ESI networks, provide emotional support, serve as role models, and assist in developing mentorship teams to meet the diverse needs of ESIs in diabetes translational research.

#### Expanding networks and career opportunities

ESIs suggest mentors provide resources for building research programs and advancing networks. Mentors can offer opportunities to attend academic, healthcare, or community meetings, allowing ESIs to gain experience and networking opportunities. Nominating ESIs as reviewers for grants or publications can also enhance their experience and networks.

#### Providing emotional support

Mentors can help normalize life events, such as raising children, and support ESIs in balancing career development with family needs. Mentors can support ESIs in the scientific process by normalizing rejection and moving forward.

#### Role modeling

Mentors should maintain open doors for ESIs to attend important meetings, exposing them to senior-level experiences. Role modeling engagement with NIH Program Officials during the grant submission process will help ESIs utilize diabetes translational research grants effectively.

#### Supporting mentorship teams

ESIs recognize that no single mentor can fulfill all their needs. Institutions should employ a mentorship team approach to address multifaceted needs, with mentors providing expertise in scientific areas, grantsmanship, career development, and emotional support.

### NIH

The NIH can support ESI growth and training by providing workshops and training institutes that foster ESI networking and diabetes translational research skill development; increasing visibility and knowledge about applying for NIH grants and options for leave; supporting community-engaged research; and promoting multidisciplinary partnerships.

#### Supporting ESI growth and training

NIH offers several opportunities for ESIs to obtain funding for their research and training goals (e.g., F-series, K-series, T-series). Continuing to support workshops and training institutes, like the National Enrichment Program, fosters networking and skill development among diabetes translational researchers. ESIs would benefit from knowing about the policies that NIH has to support leave during times when they are experiencing critical life events (e.g., childbirth, adoption, illness and/or debilitating conditions, and primary caregiving responsibilities of an immediate family member). For example, investigators can apply for administrative supplements to support additional staff, supplies, equipment, or services to enhance retention of investigators and minimize departures from the translational research workforce.

#### Increasing visibility and knowledge

To improve fairness, NIH should disseminate information about funding opportunities and grant writing strategies in accessible ways. Many “insider tips” for increasing funding chances are often shared through mentor channels, leaving some ESIs without access. NIH could offer virtual workshops and make recordings available to ensure equitable access to information. Although diabetes-specific repositories are limited, NIH-supported platforms such as NIH RePORTER, the Fogarty International Center Implementation Science Resource Repository [19], and the National Center for Advancing Translational Sciences (NCATS) Toolkit [20] provide examples of centralized resources that enhance access to training, tools, and funding information; similar models could be adapted to support diabetes translational research.

#### Supporting community-engaged research

Community-engaged research is vital for advancing diabetes translational research, but building trust and identifying health priorities can take time. NIH can support this formative work by offering funding for capacity building and recognizing community partners’ contributions.

#### Promoting multidisciplinary partnerships

Research is increasingly multidisciplinary, requiring larger teams and budgets [20,21]. NIH could offer specific funding opportunities to promote multidisciplinary collaborations with larger budget limits, encouraging ESIs to lead and participate in these teams.

#### Providing pilot funding for ESI research

Annually, NIDDK-funded CDTRs have a call for proposals for their pilot and feasibility (P&F) programs. P&F grants are designed for ESIs and often serve as a springboard into obtaining larger career development or independent investigator awards. For this special communication, we collected data from all seven currently funded CDTRs on the number of P&F grants that have been awarded since their inception in 2011 to assess the growth of the program in supporting new investigators to diabetes translational research. The CDTRs began in 2011 and have had 3 five-year funding cycles.

**Figure 1.**
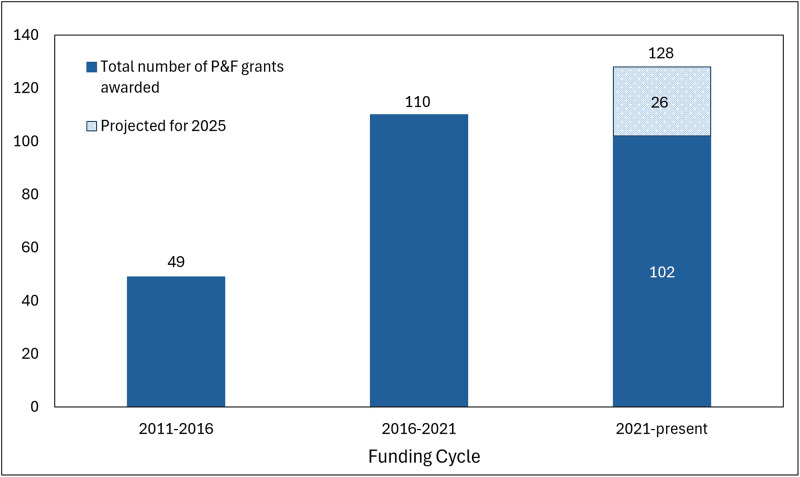
Centers for diabetes translational research pilot & feasibility awards, by funding cycle. P&F = pilot and feasibility. Figure 1 displays the number of grants funded per funding cycle. In the 2021–2025 cycle, 102 grants were awarded between 2021–2024, and an additional 26 grants are projected to be awarded in 2025, based on current trends, for a cumulative total of 128 grants in 2021–2025.

Data from the 7 CDTRs on P&F grants awarded are shown in Figure 1. Since 2011, 261 total P&F grants have been awarded by the CDTRs, and the number of grants awarded increased from 49 in 2011–2016 to 110 in 2016–2021. Thus far in 2021–2024, 102 grants have been awarded, and an additional 26 grants are projected to be funded in 2025 for a total of 128 grants in 2021–2025. In 2021, the American Diabetes Association’s partnership and financial support of these CDTR P&F grants contributed to the increase in funding over time and showcases that public-private partnerships are making an important investment in the future of diabetes translational research.

### Public health and policy leaders

Public health leaders can advocate for clear pathways for ESIs to access relevant public health data, streamline data sharing processes, and increase awareness of available datasets. They can also advocate for programs that help ESIs manage competing priorities, such as child and elder care, to support their success and well-being. Public health leaders can include diabetes translational research ESIs in strategic planning processes to ensure fair funding for diabetes translational efforts and support ESI career development, and they can provide mentorship focused on authentic community-engaged research. Policy leaders can support ESIs by enacting policies that prioritize funding for diabetes translational research, advocating for research environments, and enhancing ESI research networks.

#### Enacting supportive policies

National and institutional policy leaders can advance diabetes translational research by enacting policies that ensure ESIs thrive in their institutions and maintain successful research programs. For example, public health advocates often interface directly with policymakers and utilize research findings and perspectives from researchers to substantiate their policy briefs [22].

#### Supporting career transition programs

Policies aimed at supporting career transitions will facilitate the recruitment and retention of ESIs, creating a pipeline of multidisciplinary experts in diabetes translational research.

#### Enhancing early career research networks

Partnerships between diabetes translational research ESIs across institutions can provide valuable support. ESI networks can foster collaborations and knowledge sharing, linking ESIs to community leaders to support community-based participatory research.

### Evaluating the national enrichment program conference

One week after the conference, the DREAMS-CDTR program staff conducted an online evaluation survey using SurveyMonkey to assess attendee’s experience and generate learnings for future events (Table 2). Reminders to participate in the survey were sent at two-week intervals for 6 weeks. The survey response rate was 43% (24/56), and 88% reporting being “very satisfied” with the conference. Survey respondents also provided open-ended feedback on their conference experience; participant quotes are provided in Table 2.

**Table 2.** Conference participant satisfaction survey results

Survey response rate	24/56 (43%)
**Overall participant experience rating** “How would you rate your experience at the National Enrichment Program conference”	All respondents reported being “satisfied” or “very satisfied.” 88% of respondents reported being “very satisfied.”
**Open-ended participant feedback**	*“Thank you so much for a great conference. […] I am so grateful for the opportunity and hope that more opportunities like this are available in the future.”* *“Excellent experience. I received the best career advice.”* *“Thank you so much for hosting us - I had a fabulous day and have already followed up with 2 of the folks I ‘promised’ that I’d connect with, send stuff to, etc”* *“Great opportunity to receive feedback from the experts, gather clear directions for how to move forward. Also, great opportunity to learn about the work of others in more detail.”*
**Constructive feedback for conference improvement**	Move networking event to Day 1. Allow ESIs to connect with mentors beforehand. Let participants submit questions in advance.

While the results of the survey were overwhelmingly positive, it also provided constructive feedback on how to improve the in-person conference in the future. Ideas included incorporating the formal networking event into the first day of the conference; allowing ESIs to connect with their work-in-progress mentors prior to the in-person meeting and providing the opportunity to submit questions for conference speakers in advance. We will incorporate these learnings into the next set of NEP in-person symposia.

## Discussion

In this special communication, we summarized proceedings from the inaugural NIDDK CDTR National Enrichment Program Conference, attended by ESIs and senior faculty members from 23 institutions across the U.S. who attended. These findings underscore the multifaceted challenges and opportunities faced by ESIs in diabetes translational research and highlight the critical need for a supportive ecosystem that fosters innovation, collaboration, and career development. A limitation of these proceedings is the lack of demographic information from attendees, restricting our ability to describe participant heterogeneity. One of the most pressing challenges identified is the need for institutions to actively promote family-friendly initiatives and work-life balance that also support ESIs during critical life events. This support can take the form of flexible work arrangements, tenure clock extensions, and resources for childcare. ESIs identified rising living and childcare costs as major concerns. Magudia and colleagues found that physicians in training spent about 43% of their pretax salary on childcare during their first two years of residency [23]. However, the current childcare cost allowance for the Ruth L. Kirstein National Research Service Award (NRSA) is only $3000 per budget period starting in fiscal year 2024 [24], despite estimated childcare costs for a single child ranging from $5357 to $17,171 across the U.S. when adjusted for inflation [25]. This underscores the importance of childcare support for ESIs.

Institutions can advance ESI careers by promoting mentorship programs [26], career development, research activities, and support during critical life events. ESIs emphasized the need for increased funding opportunities, livable stipends, creative engagement spaces, and policies supporting critical life events such as family-friendly initiatives and tenure clock extensions [27]. Institutions can also enrich and foster cross-institutional collaborations to expand access to those in low-resource settings. Mentors can help develop the ESI workforce by expanding networks and providing opportunities for career advancement. A primary mentor can assist the ESI in forming a mentorship team to ensure that all their needs are met as they transition successfully to mid-career.

The NIH can continue supporting the development of a robust ESI workforce through training opportunities that foster skill development and networking, as well as funding opportunities that encourage multidisciplinary collaboration and community engagement. One example of NIH’s support for ESI networking and career development is the National Enrichment Program conference, whose proceedings guided this special communication. NIDDK’s funding of the National Enrichment Program supplement was in response to challenges identified via literature, CDTR ESI program feedback, and annual directors’ meetings. Furthermore, NIDDK’s CDTR efforts to address ESI career development in diabetes translation research include the CDTR P&F program, which has significantly increased funding for ESI investigators over the last decade. In addition to NIH funding, ESIs should also consider diverse funding sources (e.g., internal institutional seed grants, foundation awards). Continuing to support ESI-focused programs like the National Enrichment Program and other workshops geared toward ESIs (e.g., the Summer Institute for Randomized Behavioral Trials) [28], can help them learn rigorous methods and develop cross-institutional partnerships necessary for translational research. Faculty development programs have been shown to advance ESI research careers by facilitating collaboration, grant submissions, and increased involvement in national organizations [29,30]. This evidence suggests that coupling faculty development with career transition programs focused on translational research is vital for training the next generation of diabetes translational researchers. Furthermore, enacting policies that recognize the unique circumstances ESIs face, such as balancing research careers and families, can help retain them in research. For example, NIH supports leave from career development awards and offers Critical Life Event supplements to provide extra funding during critical life events [31].

These challenges and opportunities can guide academic institutions, federal funding agencies, senior mentors, and public health and policy leaders in supporting ESIs in diabetes translational research. Broadly, our insights could influence national research priorities by advocating for broader funding practices that enhance understanding of diabetes prevention and management. Our findings have implications for future policy decisions at various levels, emphasizing the importance of developing bidirectional relationships with policymakers to catalyze new partnerships between scholars and policymakers. They also highlight the need for NIDDK and other Institutes to fund diabetes translational research, particularly in retaining ESIs.

The insights from this special communication align with recommendations from a recent scientific review of Social Determinants of Health (SDOH) and Diabetes [32]. Hill-Briggs et al. recommended prioritizing research targeting SDOH through multisector partnerships. Our learnings underscore the importance of these partnerships across various sectors and guide how they can better support ESIs in diabetes translational research. Similarly, Hill-Briggs et al. recommended enhancing training priorities in multisector collaborative research approaches [32]. The findings in this special communication offer opportunities at multiple levels (e.g., program, institutional, policy) to ensure emerging scientific leaders in diabetes translation research can persist, grow careers, and address complex biomedical research challenges. While participants noted that expanded institutional and funding support is needed, detailed financial modeling and resource reallocation strategies are beyond this meeting’s scope and should be addressed in future work.

In conclusion, this Special Communication provides recommendations from ESIs that they believe will promote and facilitate greater engagement and success in diabetes translational research by strengthening and sustaining their research careers, ultimately allowing them to improve outcomes for the growing population of patients with diabetes.

## Table 1. Long description

A table with four rows and five columns. The columns are labeled ‘Area of support’, ‘Key challenges and opportunities’, ‘Existing opportunities or recommendations for needed programs informed by ESI discussions’, ‘Institutions’, and ‘Mentors’. The rows detail specific areas of support and corresponding challenges, opportunities, and recommendations. The table includes detailed text entries for each cell, providing a comprehensive overview of the support needed for early-stage investigators in diabetes translational research.

Navigate back to Table 1..
